# The emergence of the cortisol circadian rhythm in monozygotic and dizygotic twin infants: the twin-pair synchrony

**DOI:** 10.1111/j.1365-2265.2006.02706.x

**Published:** 2007-02-01

**Authors:** Rodrigo Jose Custodio, Carlos Eduardo Martinelli Junior, Soraya Lopes Sader Milani, Aguinaldo Luis Simões, Margaret de Castro, Ayrton Custodio Moreira

**Affiliations:** * Department of Pediatrics, University of Sao Paulo Ribeirao Preto, Brazil; † Genetics, University of Sao Paulo Ribeirao Preto, Brazil; ‡ Internal Medicine, School of Medicine of Ribeirao Preto, University of Sao Paulo Ribeirao Preto, Brazil

## Abstract

**Objective:**

Studies on the influence of genetic factors on the ontogeny of cortisol circadian rhythm in infants are lacking. This study evaluated the influence of twinning and the heritability on the age of emergence of salivary cortisol rhythm.

**Design and subjects:**

A longitudinal study was performed using salivary samples obtained during morning and night, at 2, 4, 8, 12, 16, 20 and 24 weeks of postnatal life in 34 infants, 10 monozygotic (MZ) and 7 dizygotic (DZ) twin pairs. Salivary cortisol was determined by radioimmunoassay (RIA). Zigosity was verified by DNA analysis of at least 13 short tandem repeat polymorphisms. Difference of the emergence of cortisol circadian rhythm, within each twin pair, the intraclass correlation coefficient and the heritability index (*h*^2^) were calculated.

**Results:**

The mean (± SEM) age of emergence of salivary cortisol circadian rhythm was similar in MZ and DZ (7·8 ± 1·0 *vs* 7·4 ± 1·3 weeks). Seven pairs showed coincidence of the emergence of cortisol rhythm. Ten pairs were not coincident; among them the within-pair difference of emergence of salivary circadian rhythm was similar in both MZ and DZ groups. The intraclass correlation coefficients were rMZ = 0·60, *P* = 0·02; and rDZ = 0·65, *P* = 0·03, respectively. The heritability index (*h*^2^) was 0·21 (ns).

**Conclusions:**

Salivary circadian rhythm appeared at the same postnatal age in MZ and DZ twin infants. Although several physiological aspects might be involved, the heritability index, obtained in the present study, suggests less genetic than environmental impact on the age of the onset of the cortisol circadian rhythm. Our data also indicated that each twin-pair show synchrony because they probably shared prenatal and postnatal environmental synchronizers.

## Introduction

The ACTH-corticosteroid rhythm is an endogenous rhythm regulated by the brain-adrenal neurohumoral circuit. The major action of the environment is to synchronize the circadian rhythms by periodic factors, such as light–dark, rest–activity and sleep–wake cycles, food ingestion and social cues.^1^–^3^ However, the effects of these synchronizers on circadian rhythm are superimposed on inherited characteristics of cortisol regulation. Salivary cortisol levels reflect free fraction of plasma cortisol concentrations. Saliva can be obtained at home, using a noninvasive and stress-free method. Therefore, salivary cortisol measurements represent a reliable tool to evaluate the hypothalamic-pituitary-adrenal (HPA) axis, including the cortisol circadian rhythm in infants and children.^4^–^8^

There are only four previous longitudinal studies using salivary cortisol. The reported mean age of emergence of the circadian rhythm was between the second and third month of postnatal life both in term and in preterm infants.^4^–^7^ Infants from multiple gestations seem to be more prone to develop postnatal adrenal insufficiency;^9^ and in sheep, adrenocortical function is delayed in twins in relation to singletons.^10^ Therefore, information on the effect of twinning on the onset of cortisol circadian rhythm in infants is still lacking.

A powerful tool to evaluate the genetic component of any trait variation is to compare the concordance of the phenotype in monozygotic (MZ) and dizygotic (DZ) twins. Comparative studies between MZ and DZ twins reported significant genetic influences on some characteristics of circadian cortisol rhythm, such as on baseline as well as on stimulated cortisol levels. Moreover, all current knowledge of the genetics of human cortisol rhythm comes from studies in adolescents and adults.^11^–^17^ There are no studies on the influence of genetic factors on the ontogeny of cortisol circadian rhythm in infants. The aim of this longitudinal study was to determine the influence of the heritability on the age of emergence of salivary cortisol rhythm by comparing MZ and DZ twin pairs.

## Subjects and methods

### Subjects

Thirty-four newborn infants (17 twin pairs) were included in this study (Table 1). None of them had brain or respiratory disorders, endocrine or metabolic problems. There was no history of glucocorticoid use by the mother or children. After DNA analysis (see below), 2 groups were formed: 10 MZ pairs (6 MZ male, 4 MZ female pairs) and 7 DZ pairs (3 DZ male, 2 DZ female and 2 DZ opposite-sex twin pairs). The gestational age evaluated by the somatic Capurro method^18^ ranged from 36 to 39 weeks (mean ± SEM 37·5 ± 0·2) in the MZ group and from 34 to 39 weeks (36·3 ± 0·4) in the DZ group. The Apgar score^19^ at 5 min was 8 for all subjects in both groups. The mean birth weight was 2499 ± 75 g in MZ and 2375 ± 89 g in DZ group. The first-born twin had higher birth weight than the second-born sibling in 8 of 10 MZ pairs and in 7 of 7 DZ pairs. Eight MZ pairs and 5 DZ pairs were delivered by Caesarean section, while the other 4 pairs were born by vaginal delivery. All subjects of the same pair were delivered by the same procedure. The lengths of stay of the babies in the hospital were 3·7 ± 0·4 and 5·3 ± 1·0 days in MZ and DZ groups, respectively. Each twin pair was living at the same home and all the infants were healthy at the time of saliva collection. The parents gave informed consent to participate in the study and the protocol was approved by the University Hospital Ethics Committee and by National Committee of Research Ethics.

**Table 1:** Clinical characteristics of the twin pairs

	Monozygotic twins	Dizygotic twins
Subjects	20 (10 pairs)	14 (7 pairs)
Gestational age (weeks)	37·5 ± 0·2	36·3 ± 0·4
Birth weight (g)	2499 ± 75	2375 ± 89
Apgar score (5th min)	9·6 ± 0·1	9·7 ± 0·1
Delivery	8 Caesarean	5 Caesarean
	2 vaginal	2 vaginal

### Zygosity testing

Blood samples from umbilical cord or exfoliated cells from oral mucosa of the newborn were obtained for zygosity testing. After the extraction, the DNA was amplified by at least 13 polymerase chain reactions (PCR) performed each with one primer pair. The PCR amplified products were detected in polyacrylamide denaturing gels stained with silver nitrate and the allele identification was achieved by comparison of the amplified fragment lengths with allelic markers. Short-tandem-repeat (STR) loci of the same lengths between pairs were indicative for monozygotic twins, while the presence of at least one different fragment lengths was indicative for the dizygotic twins.^20^ The probability of an incorrect identification of zygosity is less than 0·0001%.

### Study design

Salivary samples were obtained at weeks 2, 4, 8, 12, 16, 20 and 24 of postnatal life. Saliva samples were collected by the same physician (RJC), who took care of the babies at the hospital nursery, between 08:00 h and 09:00 h (morning sampling) and between 20:00 h and 21:00 h (night sampling) at the infant's home, as described previously.^5^,^6^ The mothers were instructed to wake up the children at least 30 min and no longer than 1 h before saliva sampling and also do not feed them in the hour prior to saliva collection.^7^ The mothers nestled their babies in their arms during the saliva sampling. Both members of each twin pair were studied simultaneously in the same room with lights on. Apparently, the infants were not distressed by the sampling procedure.

### Assays

Salivary cortisol was determined by radioimmunoassay (RIA) in 25 µl-samples of saliva.^5^,^21^ The assay sensitivity was 1·7 nmol/l. The mean intra- and interassay coefficients of variation (CV) were 8·0% and 13·7%, respectively. All samples for the same twin pair were analysed in duplicate in the same assay and the mean of the duplicate was reported.

### Statistical analysis

A circadian pattern of salivary cortisol was defined as a nighttime value less than 76% of the morning level for each subject. This value was the result of the subtraction of 24% (3 times the intra-assay CV) from the morning value taken as 100%.^5^,^6^ The daily variation must be observed for at least two consecutive weeks.

The intraclass correlation coefficient (r_i_) was used as a measure of the degree of resemblance between subjects from the same family regarding a particular variable. Intraclass correlation coefficient (rMZ and rDZ, for MZ and DZ twins, respectively) for the timing of the emergence of salivary cortisol circadian rhythm was estimated by anova.^14^ The heritability index (*h*^2^*)* was calculated from the within-group variance (${\text{S}}_{\text{P}}^{2}$) for MZ (${\text{S}}_{\text{P}}^{2}$MZ) and DZ (${\text{S}}_{\text{P}}^{2}$ DZ) pairs based on the following equation: *h*^*2*^ = ${\text{S}}_{\text{P}}^{2}$ DZ − ${\text{S}}_{\text{P}}^{2}$ MZ ${\text{S}}_{\text{P}}^{2}$ DZ. For a trait with 100% genetic determination, *h*^2^ would be closer to 1. In addition, the genetic heritability was also calculated from the intraclass correlation coefficients: *h*^2^ = rMZ − rDZ/1− rDZ.^22^ We also calculated the difference, in weeks, of the emergence of cortisol circadian rhythm, within each twin pair using the paired *t*-test and to compare MZ and DZ groups we used the *t*-test. The correlation between the birth weight and the age of emergence of cortisol rhythm was calculated by Pearson linear correlation. Data were expressed as mean ± SEM. Significance was accepted at *P <* 0·05.

## Results

There was no difference in clinical characteristics (Table 1) between MZ and DZ groups, except for a higher gestational age in MZ pairs (*P =* 0·01).

**Fig. 1:**
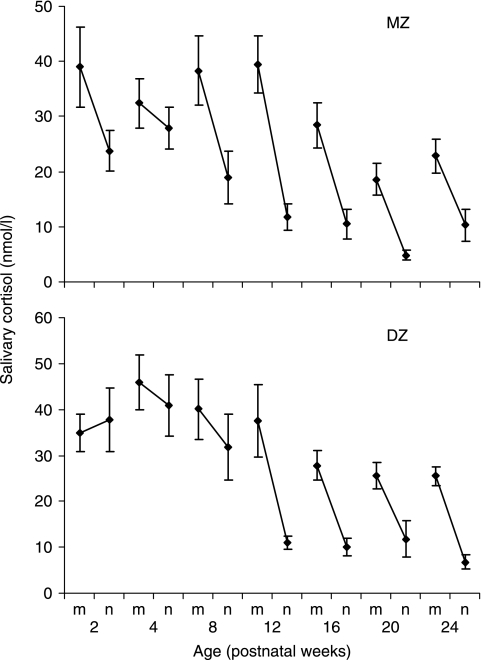
Postnatal development of salivary cortisol circadian rhythm in twin infants. Mean (± SEM) concentrations for monozygotic (MZ) and dyzigotic (DZ) groups. m, morning; n, night.

**Fig. 2:**
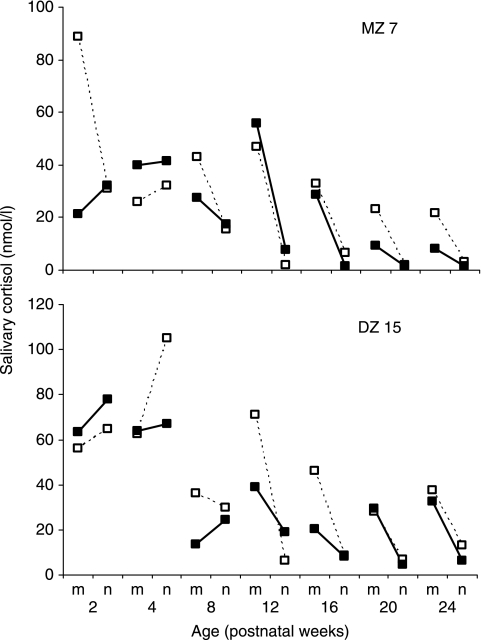
Representative examples of postnatal development of salivary cortisol circadian rhythm in monozygotic (MZ) and dizygotic (DZ) twin pairs (MZ7 and DZ15), respectively. Open and close squares represent each sibling of the same pair. m, morning; n, night.

Table 2 shows the individual age of appearance of salivary cortisol rhythm. The circadian pattern emerged in MZ and DZ groups at 2 weeks in 4 and 4 babies, at 4 weeks in 2 and 2, 8 weeks in 9 and 3, 12 weeks in 3 and 3, 16 weeks in 2 and 2, respectively. The mean (± SEM) age of emergence of salivary cortisol circadian rhythm was similar between MZ and DZ groups (7·8 ± 1·0 and 7·4 ± 1·3 weeks, NS). Irrespective of zygosity the mean age of emergence of cortisol rhythm was not different in second-born twins compared with first-born twins (7·3 ± 1·2 and 8·2 ± 1·1 weeks, NS). There was no significant correlation between the birth weight and the age of emergence of cortisol rhythm (*r* = 0·1, NS). In addition for group analysis purposes all individual data were combined. The mean values of morning and night salivary cortisol samples obtained from 2 to 24 postnatal weeks using all the absolute values for MZ or DZ twin groups showed that the cortisol daily variation emerged at the mean age of 8 and 12 weeks for MZ and DZ groups, respectively (Fig. 1). The mean values of morning salivary cortisol showed no difference among weeks 2, 4, 8 and 12, whereas they were lower at weeks 16, 20 and 24 compared to previous weeks. Regarding the mean values of night salivary cortisol, they were lower at 12, 16, 20, and 24 weeks compared to 2, 4 and 8 weeks. Five MZ and 2 DZ pairs showed coincidence of emergence of cortisol rhythm. Representative examples of coincident pairs (MZ7 and DZ15) of salivary cortisol circadian patterns are shown in Fig. 2. Ten pairs were not coincident; among them the within-pair difference of emergence of salivary circadian rhythm was similar in both MZ and DZ groups (4·8 ± 1·4 and 4·8 ± 1·0 weeks, NS). For the MZ and DZ twin pairs, the estimated intraclass correlation coefficients for the age of emergence of salivary cortisol circadian rhythm and the *F* ratio levels were rMZ = 0·60, *F* = 4·02, *P* = 0·02; and rDZ = 0·65, *F* = 4·85, *P* = 0·03, respectively. The heritability index (*h*^2^) calculated from the within-group variance was 0·21 (NS). The heritability (*h*^2^) estimated from the intraclass correlation coefficients was −0·16. *h*^2^ values < 0 were not defined and were interpreted as 0.

**Table 2:** Individual age of emergence of salivary cortisol circadian rhythm, in weeks, in monozygotic and dizygotic twin pairs

Monozygotic pairs		Dizygotic pairs	
	
Subjects	Weeks	Subjects	Weeks
1*a*, 1*b*	4, 2	11*a*, 11*b*	4, 8
2*a*, 2*b*	16, 8	12*a*, 12*b*	16, 8
3*a*, 3*b*	8, 8	13*a*, 13*b*	2, 2
4*a*, 4*b*	8, 8	14*a*, 14*b*	12, 16
5*a*, 5*b*	12, 8	15*a*, 15*b*	12, 12
6*a*, 6*b*	12, 12	16*a*, 16*b*	8, 2
7*a*, 7*b*	8, 8	17*a*, 17*b*	4, 2
8*a*, 8*b*	8, 16		
9*a*, 9*b*	2, 2		
10*a*, 10*b*	4, 2		

*a* and *b* represent the first and the second born twins from each pair, respectively.

## Discussion

The first purpose of this study was to examine the effects of twinning on the ontogeny of the salivary cortisol circadian rhythm in infants. Our results indicate that the mean age of the appearance of the ‘adult-like’ pattern of circadian variation of cortisol were 7·8 ± 1·0 and 7·4 ± 1·3 postnatal weeks in MZ and DZ twin groups, respectively.. Individual data showed that the majority of the infants established their cortisol rhythm as early as at 2, 4 or 8 postnatal weeks. These data on the emergence of cortisol circadian rhythm in twins are similar to those previously observed in our laboratory in term^5^ and preterm^6^ babies from singleton gestations, applying similar methodologies. It is important to point out that the salivary cortisol daily variation emerged in both groups, as a whole, at 8 and 12 weeks for MZ and DZ groups, when all the absolute values for each twin group were combined. This discrepancy between MZ and DZ groups might be explained by a high variability among subjects. Our data also showed that the mean daily concentration of salivary cortisol is greater in the first two months of postnatal life than in subsequent months, as previously described.^4^ The age of appearance of cortisol rhythm is still matter of discussion. The acquisition of this rhythm varies from 2–3 months^4^,^7^,^23^,^24^ to 9 months.^25^ The lack of consensus from these studies can be ascribed to different definitions of circadian rhythm, sampling times and methods of data analysis. Therefore, the interpretation of our data should take into account all these methodological aspects, apart from the fact that the present data is limited to the onset of cortisol circadian rhythm. Finally, it should be emphasized that the cortisol circadian mechanisms continue to mature in infant and toddler periods in human.^26^

The adrenocortical function may be impaired in infants from multiple gestations.^9^ Previous study in healthy adult twin pairs demonstrated that the sibling who was born lighter showed a lower basal serum cortisol levels compared to the heavier sibling.^27^ This can be ascribed to the intrauterine environment, which may be considered suboptimal for twin fetuses, mainly for the second born of the twin pair, as compared with that of singletons.^10^,^28^ Our data indicate that the effects of twinning do not have consequences on the postnatal emergence of cortisol circadian rhythm even for the second-born twin.

Several clock genes and their cognate proteins have been described and create molecular cycles that approximate the 24-h environmental cycle.^29^ The overt rhythms result from a complex interaction between the genetic output of the endogenous circadian pacemaker and the periods of the environmental synchronizers.^1^–^3^ Therefore, the second and main purpose of this study was to obtain insights on the genetic and environmental influences on the ontogeny of cortisol circadian rhythm in infants. The different degree of genetic relatedness between MZ and DZ twin pairs was used to estimate the relative contributions of genes and environment to the phenotypic expression of cortisol circadian rhythm in twin infants. There are few previous comparisons of some characteristics of the cortisol circadian rhythm in adolescents and adult twins^13^,^15^–^17^ but none focused their attention on the ontogeny of such rhythm. In the present study, we observed that the estimated heritability index for the age of emergence of cortisol rhythm was low and nonsignificant (ns). Our data does not support the assumption of a considerable genetic effect on the age of onset of salivary cortisol circadian rhythm. Genetic influences have been demonstrated for the cortisol levels during the awakening period or for the timing of the nocturnal nadir, but not for cortisol levels during the day or the 24-h mean.^13^,^17^ Recently, Kurina *et al*. (2005) reported two novel loci that influence morning cortisol levels in women.^30^ It is possible that distinct genes control some characteristics of the cortisol rhythm at different times of the day,^13^,^16^,^29^ while other features, such as the emergence of cortisol diurnal cycle, are predominantly influenced by environmental factors that overshadow any genetic influence on individual differences.

We also found that both the MZ and DZ intraclass correlation coefficients were similar in size and significance. These data indicate that there are within-pair common environmental or familial factors influencing the appearance of cortisol circadian rhythm in infants. The ontogenetic changes in the developing of cortisol circadian rhythm may reflect prenatal and postnatal concurrent maturation of the suprachiasmatic nucleus (SCN) and its specific neuroanatomical pathways that mediate the effects of each particular photic and nonphotic synchronizer agents.^2^,^3^ The circadian system seems to be functional in fetal life. The main entrainment factors to the programming of the fetal circadian cycles are received from maternal circadian inputs.^31^–^33^ Therefore, twin-fetuses shared prenatal synchronizing factors. Additionally, the putative mechanism of tactile communication between twins *in utero*, irrespective of the zygosity^34^ may also contribute to the marked intrapair correlation of the age of the emergence of cortisol circadian rhythm in twins. Therefore, subsequent postnatal similarities within-pair may originate *in utero*.

At birth, there is no circadian rhythm of sleep, body temperature, melatonin production and cortisol secretion.^35^–^37^ These rhythms in singleton babies seem to occur at much earlier postnatal age than previously reported.^5^,^6^,^36^ In the present study, for the first time, we demonstrated that salivary cortisol rhythm appears at the same postnatal age in twin babies as singleton infants. In human, the period of light-dark cycle is a dominant environmental synchronizer to entrainment of the circadian rhythms^2^ but the mother–infant interactions and household's social routine remain as nonphotic synchronizer factors.^24^,^36^,^38^,^39^ Therefore, in addition to common prenatal synchronizing factors, each twin-pair also shares postnatal life environment, including twin–twin interactions. Taken together, all of these common environmental factors contribute to the within-pair synchrony on the age of the emergence of cortisol rhythm in MZ and DZ twins.

In conclusion, the present study showed that the salivary circadian rhythm appeared at the same postnatal age in MZ and DZ twin infants. Although several physiological aspects might be involved, the heritability index, obtained in the present study, suggested less genetic than environmental impact on the age of the onset of the cortisol circadian rhythm. Our data also indicate that each twin-pair show synchrony among their emergence of cortisol circadian rhythm probably because they shared prenatal and postnatal environmental synchronizers.
